# The Reported Use of Tongue-Ties and Nosebands in Thoroughbred and Standardbred Horse Racing—A Pilot Study

**DOI:** 10.3390/ani11030622

**Published:** 2021-02-26

**Authors:** Dominic Weller, Samantha Franklin, Peter White, Glenn Shea, Kate Fenner, Bethany Wilson, Cristina Wilkins, Paul McGreevy

**Affiliations:** 1 Sydney School of Veterinary Science, Faculty of Science, University of Sydney, Sydney, NSW 2006, Australia; p.white@sydney.edu.au (P.W.); glenn.shea@sydney.edu.au (G.S.); kate@kandooequine.com.au (K.F.); bethany.wilson@sydney.edu.au (B.W.); paul.mcgreevy@sydney.edu.au (P.M.); 2 School of Animal and Veterinary Sciences, University of Adelaide, 1454 Mudla Wirra Road, Roseworthy, SA 5371, Australia; sam.franklin@adelaide.edu.au; 3 Saddletops Pty Ltd., P.O. Box 557, Gatton, QLD 4343, Australia; editor@horsesandpeople.com.au

**Keywords:** horse, equitation science, tongue-ties, nosebands, welfare, safety, tack, welfare

## Abstract

**Simple Summary:**

Tongue-ties (TTs) are commonly used in racing to restrain a horse’s tongue to aid a rider’s/driver’s control of the horse and optimise upper airway function. Nosebands (NBs) may also be employed for similar purposes. This article reports on a survey that asked people involved in Thoroughbred (TB) and Standardbred (SB) racing whether they used TTs and NBs and, if they did, the reasons for their use, the preferred design of device, the devices’ perceived effectiveness at achieving the respondents’ desired outcome(s), any complications due to their use and whether or not these complications altered their decision to use a particular type of TT or NB. A total of 112 participants involved with TB and SB racing answered TT questions. It revealed that respondents who used TTs believed them to be *very* or *extremely effective* at preventing the tongue from moving over the bit and improving upper airway function. Both physical and behavioural complications due to the use of a TT were reported. The likelihood of a respondent reporting a complication due to TT use increased with every minute of reported application and a nine-minute increment in the reported duration of application doubled the odds of a respondent reporting a behavioural complication. The findings of this study should be considered only as those of a pilot study and should be interpreted with caution due to the small number of responses.

**Abstract:**

This article reports on the results of a survey of racehorse trainers (*n* = 112) outlining the reasons for tongue-tie (TT) and noseband (NB) use by Thoroughbred trainers (TBTs) (*n* = 72) and Standardbred trainers (SBTs) (*n* = 40). The study also investigated the reported effectiveness of TTs and possible complications arising from their use. Tongue-tie use was reported by 62.5% (*n* = 70) of racehorse trainers. The reasons for TT use varied between TBTs and SBTs. For TBTs, the most common reason for TT use was to prevent or reduce airway obstruction (72.3%, *n* = 34), followed closely by to prevent or reduce airway noise (55.3%, *n* = 16). Standardbred trainers assigned equal importance for TT use [to prevent or reduce airway obstruction (69.6%, *n* = 16) and to prevent the horse from moving its tongue over the bit (69.6%, *n* = 16)]. Tongue-ties were considered significantly less effective at improving performance than at reducing airway obstruction and preventing the tongue from moving over the bit (*t* = −2.700, *p* = 0.0007). For respondents who used both TTs and NBs, there was a mild to moderate positive association between the reasons for using TTs and NBs. Of the 70 TT-using respondents, 51.4% (*n* = 36) recorded having encountered either a physical or behavioural complication due to TT use, with redness/bruising of the tongue (20.0%, *n* = 14) being the most common physical complication reported. Duration of use influenced the risk of observing complications. The likelihood of a respondent reporting a behavioural complication due to TT use increased with every minute of reported application and a nine-minute increment in application period doubled the odds of a respondent reporting a complication. Tightness was a risk factor for physical complications: Checking TT tightness by noting the tongue as not moving was associated with increased reporting of physical complications (OR = 6.59; CI 1.1–67.5). This pilot study provides some insight into how and why TTs are applied by some racehorse trainers, and the potential risks associated with their use. A further study of a larger cohort is recommended because these results are valid for only the 112 trainers who responded and cannot be generalized to the equine industry.

## 1. Introduction

The long history of horseracing has seen the introduction of numerous devices designed to increase control of horses and generally improve their performance. Tongue-ties (TTs) and nosebands (NBs) are common examples. Tongue-ties are bands or straps, made of elastic, nylon or leather, that are wrapped around the tongue of a horse, affixing it to the mandible [1]. They have been recommended since at least the 1800 s, as a conservative treatment for upper airway obstruction [2], in particular dorsal displacement of the soft palate (DDSP) [3]. This condition involves the free caudal margin of the soft palate becoming displaced from its normal sub-epiglottic position during exercise, resulting in an obstruction to airflow through the aditus laryngis [1]. This can lead to impaired athletic performance due to reduced oxygen supply to the exercising muscles [1]. Dorsal displacement of the soft palate is one of the most common forms of dynamic upper airway collapse affecting racehorses [4,5]. Studies have estimated that up to 20% of racehorses may be affected by the condition [6,7]. However, the exact prevalence is difficult to determine since a definitive diagnosis requires an exercising endoscopic examination.

It has been suggested that TTs may prevent DDSP by stabilising the nasopharynx and preventing caudal retraction of the tongue and hence the larynx, which may subsequently become dislocated from the ostium intrapharyngeum [8]. In resting horses, the application of a TT has been reported to alter the position of the lingual process of the basihyoid bone [9]. However, studies of normal horses examined during treadmill exercise suggest that TTs do not alter upper airway mechanics [10]. Furthermore, few studies have assessed objectively the efficacy of the TT to prevent DDSP in affected horses, while those that have used exercising endoscopy found that TTs were effective in only small numbers of horses [11,12,13]. Tongue-ties act to restrict the tongue’s movement within the oral cavity so that the horse cannot move the tongue over the bit. 

Concerns about the effect of TTs on equine welfare have led to their use being banned in equestrian disciplines, by the Fédération Equestre Internationale (FEI) since 2004 and, for racing, in Germany since 2018 [14]. More recently, Racing Australia banned the use of nylon stocking TTs in TB racing, although other materials are still allowed [15]. Tongue-ties are also still widely used in racing elsewhere in the world, such as in the UK where reportedly 5% of horses race with a TT [16]. 

Another piece of equipment commonly used to control horses is the NB. Nosebands are available in many different designs but are generally an addition to the bridle that fits around the bridge of the nose, encircling the mandible and maxilla. They are widely used across a range of equestrian disciplines and, in some cases, may be restrictive enough to prevent the horse from opening its mouth [17]. In non-racing contexts, NBs are used for a variety of reasons, notably to improve rider/driver safety, by reducing evasion of the bit and making the horse easier to steer and decelerate in response to rein tension, at least in the short-term [18]. Some NB types (such the Grackle or figure-of-eight) are sometimes applied to restrict a horse’s ability to open its mouth and prevent the occurrence of DDSP and have been reported to be more effective than TTs for this purpose [13]. 

This report reflects the dual purpose of a survey presented to a variety of respondents. Previously, the current researchers explored only the use of NBs across equestrian sports and racing [19] because the initial response rate to TT questions was considered poor. Broadly, our first report showed that NBs were used equally for anatomical, consequential and passive reasons [19]. The study also identified issues with the preferred sites for checking NB tightness (with many respondents checking at sub-optimal locations such as at the horse’s cheeks), as well as identifying that so-called crank nosebands were particularly problematic, with their use increasing the likelihood of respondents reporting a complication [19]. As that study had sufficient data for only NB analysis, the survey was kept online until more respondents using TTs had participated. Furthermore, given that TTs are banned in most equestrian disciplines, only a negligible number of polo/polocrosse trainers responded to the current survey and TTs are most widely used in racing, the authors decided to focus the current study to their use in racing. 

The aim of the current study was to explore the reported reasons for TT use in TB and SB racing, the estimated effectiveness of these devices at achieving those reasons and the reported prevalence of complications associated with their use. The use of TTs in combination with NBs was also a focus of the current study.

## 2. Materials and Methods 

Approval from the Human Research Ethics Committee of the University of Sydney was obtained for this study (Approval number: 2018/305).

A questionnaire was developed in RedCAP [20]. Respondents were asked about whether or not they used TTs. Additional questions related to the types of TTs used and rationale for their use, the fitting of TTs and potential complications due to their use. A full transcript of the survey can be found in the Supplementary Materials File S1. The use of NBs in racing and other equestrian disciplines has been reported separately elsewhere [19]. However, due to a low response rate for respondents reporting on TT use at the time of data collation for that study, the survey remained online, and additional efforts were made to increase awareness of the survey among racehorse trainers. 

The branching logic of the survey software [20] allowed questions to appear only as they applied to certain participants. This meant that the same survey could be dispersed across various interest groups involved with horses. Questions relating to TTs were presented to only the respondents involved in TB and SB racing, as well as polo/polocrosse respondents. However, the number of responses received from polo/polocrosse respondents were too low for any statistical analysis to be performed and their data were dropped from the study. 

Respondents were asked to rank the most important reasons (with a maximum of five) for their use of TTs from the following list of ten options: *to improve the rider’s/driver’s ability to decelerate the horse*; *to improve the rider’s/driver’s ability to steer the horse*; *to prevent or reduce airway obstruction*; *to prevent or reduce airway noise*; *to improve performance in competition*; *to prevent the horse moving its tongue over the bit*; *the bit I use requires the use of a TT*; *a veterinarian told me that I needed to use one*; *to align with rules of the sport*; and *other* (with selecting *other* prompting the respondent to elaborate). For a subset of these reasons, they were also asked to comment on the TT’s efficacy in achieving the stated purpose (*extremely effective*, *very effective*, *effective*, *somewhat effective*, *not at all effective*). Respondents were asked how long (in minutes) the TT was usually left on, and whether they noticed any undesirable physical effects or behavioural complications from its use, for which the ten possible options were: *tongue swelling during or after application*; *redness/bruising/discolouration of the tongue*; *cuts on the tongue*; *soreness of the tongue or of the lower jaw*; *nerve damage to the tongue (causing the tongue to hang out of the mouth)*; *reduced appetite*; *dropping food*; *behavioural signs of anxiety or distress*; *difficulty fastening the TT*; and *other*. Respondents were also asked to specify how they ensure the TT is applied correctly, by selecting one option from the following list of five: *it doesn’t slip forward*; *it stops the tongue moving completely*; *the horse cannot remove the TT*; *the tongue is held within the mouth*; and *other*. Respondents were asked what type of TT was used, selecting options from the following list of six: *stocking*; *elastic*; *leather*; *cotton*; *cable-ties (also known as zippy grips)*; and *other*. Lastly, respondents were asked whether they had used any alternatives to TTs by selecting options from the following list of seven: *W bit (such as the Serena Song Dexter Racing Bit)*; *tongue clip/tongue depressor*; *miracle bit*; *winning tongue plate bit*; *ported bit*; *bitless bridle*; and *other*.

### 2.1. Contact List Creation and Distribution 

A database of breed and discipline associations, online magazines and individual TBTs and SBTs from both Australia and other English-speaking countries was compiled from web searches that included the Australian Yellow Pages website, Australian Racehorse Directory, and state racing bodies/associations. Respondents were encouraged to share the survey with their networks with the intention of further disseminating the survey. An article in the December issue of the Australian equestrian journal, *Horses and People* magazine, was commissioned to raise awareness among its readers. Follow-up emails were circulated two months later. A Facebook page with details of the survey was posted to boost awareness of the survey through social media. This page and its contents were hosted on both the University of Sydney’s *Veterinary Science* and the University of Adelaide’s *Equine Health and Performance Centre* Facebook pages. Pamphlets were also distributed by hand during the Equitana conference in Melbourne in November 2018. The survey remained online for nine months, from 28/8/18–28/5/19, three months longer than the previous study [18]. A participation information statement directed participants to answer the questionnaire once only. 

### 2.2. Data Analysis

Data were analysed using the “MASS” package [21] in the R statistical software environment [22] version 3.6.1. Percentages of respondents who used TTs between the two racing codes, and percentages of respondents using particular materials as TTs, were compared through χ^2^ tests [21,22]. Descriptive frequencies were identified in Microsoft Excel.

#### 2.2.1. Reasons for TT and NB Use

For analysis, reasons for TT and NB use were grouped as “anatomical”, “consequential” and “passive”. For TTs, the anatomical reason was *to prevent the horse moving its tongue over the bit.* The consequential reasons were: *to improve the rider’s/driver’s ability to decelerate the horse; to improve the rider’s/driver’s ability to steer the horse; to prevent or reduce airway obstruction; to prevent or reduce airway noise;* and *to improve performance in competition*. The passive reasons were: *most people in my sport use them; the bit I use requires the use of a TT; a veterinarian told me that I needed to use one;* and *to align with the rules of the sport.*

The breakdown of reasons for NB use were reported in detail in a previous study [19].

#### 2.2.2. Effectiveness of TTs

The respondents’ report of the perceived effectiveness of TTs in achieving the intended outcome was measured on an ordinal scale and evaluated using a parallel log odds ordinal logistic regression model. The five-point efficacy scale was set as the dependent ordinal variable with the breed of horse (SB or TB) and the usage reason (anatomical and consequential reasons only) being rated for effectiveness (as well as the interaction between breed and usage reason) considered as explanatory variables. Because the interaction term was not significant (Likelihood ratio statistic = 1.270, *p* = 0.866), this interaction term was dropped from the model and a reduced additive model was used. Additionally, due to low numbers of ratings, *to improve the rider’s/driver’s ability to decelerate the horse* and *to improve the rider’s/driver’s ability to steer the horse* were dropped. Model suitability was assessed by graphical examination of surrogate-based residuals (calculated by the SURE package) and the proportional log odds assumption using the Brant package.

#### 2.2.3. Risk Factors for Complications of TT Use

Risk factors for physical and behavioural complications of TT use were modelled using binary logistic regression, with a binary variable coding for whether the user reported observing a complication set as the dependent variable. Potential risk factors included the breed of the horse, the reasons selected for using a TT, the method used for checking TT tightness, the material from which the TT was made, the average time (in minutes) a TT was left on, the number of horses the respondent uses a TT on, the age, gender, and horse-work experience of the respondent.

Preliminary univariate logistic regression analyses were performed on each potential risk factor, and those risk factors with a χ^2^ *p*-value < 0.25 in an ANOVA of the univariable analysis were passed to multivariable analysis. For physical complications, potential risk factors passed to multivariable analysis were: Breed, a vet recommendation for using a TT, reduction of airway noise as a reason for using a TT, the bit requiring use of a TT, tightening the TT until tongue is immobile, using a TT to prevent tongue from moving over the bit, the number of minutes a TT was reported left in place, and use of a stocking TT. A full additive logistic regression model of all potential risk factors was then run, and then compared to a reduced model with the least significant risk factor removed, until a χ^2^ on the difference in residual deviance returned a *p* of <0.05, or the Akaike Information Criterion (AIC) rose. The final model for physical effect risk factors included breed, a vet recommendation for using a TT, the bit requiring use of a TT, tightening the TT until tongue is immobile and the number of minutes a TT was reported left in place. The AIC of this model was 77.55.

For behavioural complications potential risk factors passed to multivariable analysis were number of horses worked with TT, use of TT to prevent airway obstruction, number of horses worked, use of TT to help steer horse, breed (and therefore type of racing), checking horse can’t remove TT as tightening method, gender of respondent, use of a stocking TT, use of TT to help decelerate the horse, use of TT to reduce airway noise, and the number of minutes a TT was reported left in place. The final model for behavioral effect risk factors included reduction of airway obstruction as a reason for using a TT, number of horses in training, improved control of steering as a reason for using a TT, breed, checking horse cannot remove TT as tightening method, gender of trainer, use of a stocking TT, reduction of airway noise as a reason for using a TT and number of minutes a TT was reported left in place. The AIC of this model was 63.18.

#### 2.2.4. Relationship between TT and NB Use

A Fisher’s exact test, using the “fisher.test” function of R, and post hoc pair-wise tests, using the “prop.test” function, were used to examine the relationships between noseband and TT use among the respondents (with the differences in the types of TT used by TBTs and SBTs being similarly processed). This was followed by post hoc χ^2^-based proportion tests for each type of noseband. To explore whether nosebands and TTs were used for the same purpose, the Yule-Y coefficient of colligation was calculated between paired reasons for noseband use and TT use. The reasons most similar to each other from both sections of the survey were paired and analysed through AIC. They were: *to improve the rider’s/driver’s ability to decelerate the horse; to improve the rider’s/driver’s ability to steer the horse; to prevent or reduce airway obstruction; to prevent or reduce airway noise; to improve performance in competition; to prevent the horse moving its tongue over the bit; most people in my sport use them;* and *a veterinarian told me that I needed to use one*. The strength of association was assessed on a scale from −1 to +1. An association was considered weak if the correlation was between 0 to +/−0.3, a moderate association was between +/−0.3 to +/−0.6, and a strong association was between +/−0.6 to +/−1 [23]. 

## 3. Results

### 3.1. Respondent Demographics

Overall, 112 respondents involved in racing answered the question *do you currently train/race any of your horses with a tongue tie*? Of these 112 respondents, most were from Australia (65.2%, *n* = 73). Others were from New Zealand (13.4%, *n* = 15), Sweden (8.0%, *n* = 9), UK (2.4%, *n* = 3), USA (1.8%, *n* = 2), other countries (6.4%, *n* = 7) and 2.7% (*n* = 3) undisclosed. The breed distribution included 72 (64.3%) TBs and 40 (35.7%) SBs. The ages of respondents were distributed as follows: 18–25 (7.1%, *n* = 8), 26–35 (18.8%, *n* = 21), 36–45 (17.9%, *n* = 20), 46–55 (24.1%, *n* = 27), 56–65 (13.4%, *n* = 15), 66–75 (8.0%, *n* = 9), 76–85 (9.8%, *n* = 11), and 86–95 (0.9%, *n* = 1). The average number of horses trained by all respondents was 22.8 (standard deviation +/− 57.9) and 62.5% (*n* = 70) of racehorse trainers stated that they used TTs. For respondents who did not use a TT (*n* = 41), most (*n* = 34, 82.9%) reported that there was no need, 10 (24.4%) reported that the horse appeared in pain (from using the TT), 3 (7.3%) stated that TTs were not allowed in their sport, and 4 (9.8%) selected other reasons, without further elaboration. There was no significant difference in TT use between TB and SBs (TB = 47/72 (65.34%); SB = 23/40 (57.5%); χ^2^ = 0.3733; *p* = 0.54).

### 3.2. Preferred Types of TTs

The distribution of TT types are reported in Table 1. All TBTs (*n* = 47) and SBTs (*n* = 23) reported using one or more types of TTs. Stocking TTs were most favored by TBTs (45.2%, *n* = 28) with elastic TTs most favored by SBTs (42.9%, *n* = 12). There was no significant difference between the types of TTs used by TBTs and SBTs (*t* = 1.258, *p* = 0.24).

### 3.3. Reasons for TT Use

No significant differences in the reasons for TT use were found between TB and SB racehorse trainers (Table 2). The most common reasons offered for TT use by TBTs, in order, were: *to prevent or reduce airway obstruction*, *to prevent or reduce airway noise*, and *to prevent the horse from moving its tongue over the bit*. Among SBTs, the most common reasons offered for TT use, in order, were: *to prevent or reduce airway obstruction*, *to prevent the horse moving its tongue over the bit* and *to prevent or reduce airway noise*.

Several alternatives to TTs were also reported to be used by racehorse trainers who reported using TTs. The *Serena song Dexter racing bit* was the most common alternative (28.8%, *n* = 21), *tongue clip/depressor* the next (21.9%, *n* = 16), then *winning tongue plate bit* (20.5%, *n* = 15), *bitless bridle* (11.4%, *n* = 8), *ported bit* (10.0%, *n* = 7), and *miracle bit* (6.8%, *n* = 5). Note that trainers could select more than one type of TT alternative.

### 3.4. Perceived Efficacy of TT Use among Racehorse Trainers

The effectiveness of TTs at *preventing or reducing airway obstruction* was scored by 50 respondents (69.6% (*n* = 16) SBTs and 72.3% (*n* = 34) TBTs). The modal response for both TBTs and SBTs was that it was *very effective*. For TBTs (61.7%, *n* = 29) and SBTs (43.5%, *n* = 10) who rated the effectiveness for *preventing or reducing airway noise*, the modal response for both was *very effective*. For TB (44.7%, *n* = 21) and SB (69.9%, *n* = 16) respondents who rated the effectiveness for *preventing the tongue from moving over the bit*, the modal response for both was *extremely effective*.

The overall perceived effectiveness of the TT as a piece of racing equipment was assessed by an ordinal logistic regression of the 168 effectiveness scores given to the TT for the purpose(s) for which each racing respondent employed it. This modelling showed no significant difference in the perceived effectiveness of TTs by TBTs and SBTs (t = 0.44, *p* = 0.66) corrected for the purpose for which it had been employed. Compared to the most commonly employed purpose of *preventing or reducing airway obstruction*, respondents who used TTs for *preventing the tongue from moving over the bit* found them significantly more effective (*t* = 2.624, *p* = 0.009) and those who used TTs to *improve performance* found them significantly less effective (*t* = −2.700, *p* = 0.007).

### 3.5. Duration of TT Application and Methods for Checking TT Tightness

On average, from *n* = 65 respondents, TTs were left in place for 24.3 min (standard deviation +/−20.29). The most common methods for checking tightness were *it (the TT) doesn’t slip forward* (55.7%, *n* = 39) and *the tongue is held within the mouth* (54.3%, *n* = 38), followed by *the horse cannot remove the TT* (41.4%, *n* = 29), and *it stops the tongue moving completely* (15.7%, *n* = 11).

### 3.6. Associations between TT and NB Use

The level of NB use reported by respondents is outlined in Table 3.

There was no statistical difference between a TT-using respondent and non-TT-using respondent for using a NB (χ^2^ = 1.09, *p* < 0.05). Grackle NBs (34.4%, *n* = 21) and plain cavesson NBs (34.4%, *n* = 21) were the most commonly reported type of NB used by TT users, followed by Hanoverian NBs (18.0%, *n* = 11), drop NBs (8.2%, *n* = 5), and Micklem NBs (1.6%, *n* = 1). There was a significant association between TT use and the type of NB used (*p* < 0.01). Respondents who used TTs were more likely to use Grackle/figure-of-eight NBs (prop.test χ^2^ = 3.8765, df = 1, *p* = 0.048) than respondents who did not use TTs.

### 3.7. Are the Reasons for TT Use Reflective of the Reasons Stated for NB Use in Racing?

There was a mild to moderate positive association between the reasons that TTs and nosebands were employed among those respondents who reported using both items of gear (Table 4).

### 3.8. Complications Associated with TT Use

**Figure 1:**
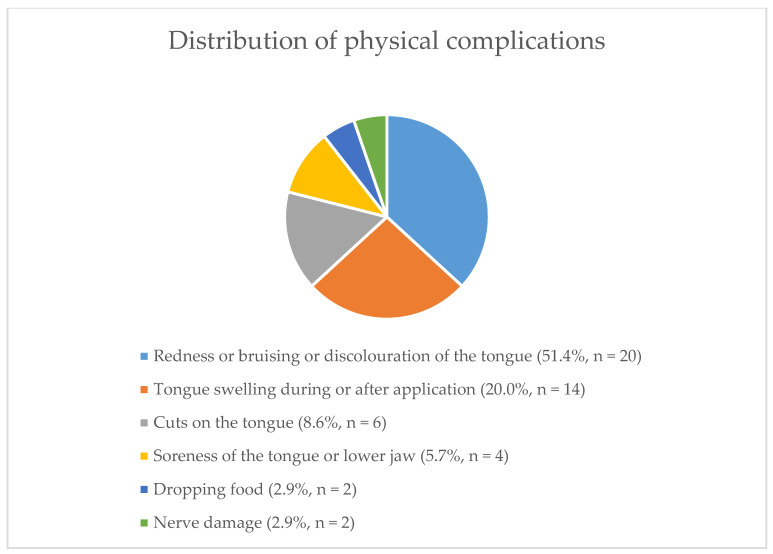
The distribution of physical complications reported by respondents (*n* = 36).

Physical complications reported from TT use are summarised in Figure 1.

Out of 70 respondents, 37 reported one or more complications due to TT use. More physical complications were reported by TBTs (53.2%, *n* = 25) than SB respondents (21.7%, *n* = 5) [Odds Ratio (OR) = 15.6; 95% CI = 3.0–156.9]. Checking TT tightness by noting the tongue as not moving was associated with increased reporting of having observed physical complications (OR= 6.59; CI 1.1–67.5). Other risk factors for physical complications did not reach statistical significance. 

**Figure 2:**
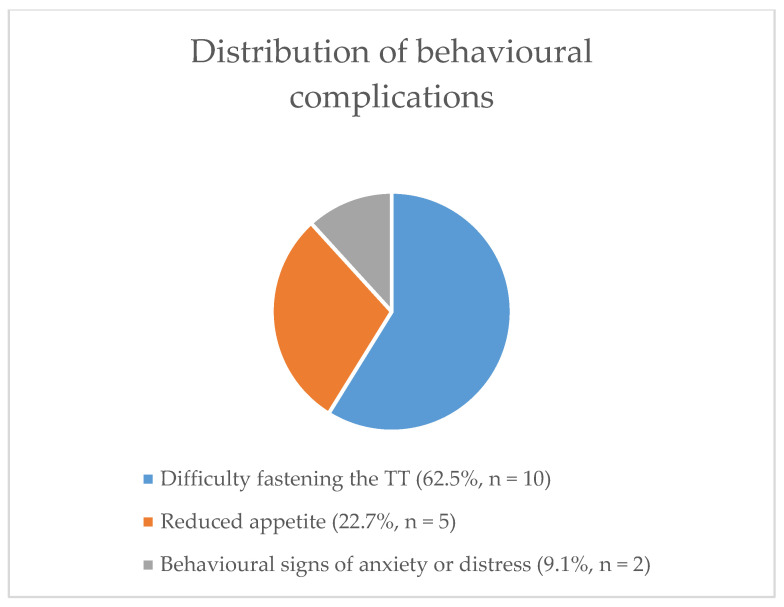
The distribution of behavioural complications reported by respondents (*n* = 16).

Behavioural complications reported from TT use are summarised in Figure 2. Use of TT for the purpose of improving steering was associated with observing a behavioural complication (*z* = 2.564, *p* = 0.010), with 4/9 (44.4%) respondents who reported using TTs for this use reporting behavioural complications, compared with 22.9% of TT respondents overall. Use for prevention of airway obstruction was also a risk factor (OR = 21.0; CI 1.8–877.7), as was checking tightness by tightening the TT to the point it could not be removed (OR = 9.4; CI 1.3–120.3) and use of a stocking TT (OR = 29.7; CI 2.5–884.0). As with physical complications, TBs were at relatively more risk than SBs (OR = 12.4; CI 1.8–146.0). Finally, the duration of time that TTs were applied for was associated with behavioural complications (*t* = 2.587, *p* < 0.01) with an OR = 1.08 (CI 1.02–1.17) per minute, or with odds approximately doubling after 8–9 min.

## 4. Discussion

This pilot study reports on the use of TTs and NBs in racing horses and reveals some of the factors associated with their use, particularly in Australia and New Zealand. These findings should be assessed with caution, due to the low response rate, so a larger study is needed to substantiate the current findings. Additionally, only some commentary will be provided regarding NBs as their use was assessed in depth in a previous study [19]. Of the 112 racehorse trainers who answered TT questions, 62.5% (*n* = 70) reported that they used TTs. This percentage is comparable to Findley et al. [24] who revealed 85% (*n* = 455) of Australian SB respondents used TTs. Barakzai et al. found that the prevalence of TT use in the UK was 5% (*n* = 377), based on data from 60 randomly selected race meetings with 7536 individual race starters [16]. 

In horse racing, the overall use of TTs has been largely attributed to preventing the tongue from moving over the bit and to their putative function as a conservative treatment for upper respiratory tract obstructions, specifically DDSP [1]. The apparent popularity of TTs within the current small sample may reflect the protracted history of using these devices in racing [7]. In the current study, among both TBTs and SBTs, *reducing airway obstruction* was the most common reason for the use of TTs, with SBTs in particular also electing to use TTs *to prevent the tongue from moving over the bit*. In comparison, Findley et al. reported on 535 harness racehorse trainers and found that only 37% (*n* = 134) used a TT for suspected upper airway obstruction and that preventing the tongue from moving over the bit was the most common reason for use (78%, *n* = 387) [24].

The reported emphasis on reducing upper airway obstruction and noise may provide a reason for the popularity of TT use among racehorse owner/trainer respondents. Conditions such as DDSP are relatively common, with an estimated 20% of racehorses thought to be affected by this condition [11] which significantly reduces oxygen uptake during exercise, thereby decreasing performance [1]. The perceived benefit of optimising airflow in a horse infers that many respondents are tying their horses’ tongues to improve overall performance. However, in the current study, this option was selected by less than half of TBTs (42.6%, *n* = 20) and SBTs (39.1%, *n* = 9). It should be noted that, to date, very few studies have investigated the efficacy of TTs [11]. The few small studies that used exercising endoscopy to objectively assess the ability of the TT to prevent DDSP found them to be ineffective in most cases [11,13]. This raises questions as to why respondents, on average, reported that TTs were *very effective* particularly at *preventing or reducing airway obstruction* (*n* = 50) and *preventing or reducing airway noise* (*n* = 39). This could be explained, in part, due to a possible placebo effect in assuming that a TT is effective at improving the airways of the horse, or perhaps that trainers who found TTs to be useful were more likely to report on their use and, as such, might contribute some respondent bias. That said, the possible benefits of TTs were shown in the study by Barakzai et al. that revealed 56.5–59.3% of race starters “improved their earnings” when they wore a TT consistently [16]. Overall, it is clear that further studies including larger numbers are warranted to further investigate the efficacy of TTs.

According to Findley et al. 78% (*n* = 387) of respondents used TTs to prevent the tongue from moving over the bit [24]. The current study’s results are similar in magnitude to those findings in that most SB owner respondents (69.9%, *n* = 16) report the use of TTs to prevent the horse from moving its tongue over the bit as well as improving rider control over the horse [24]. Control of horses is a pivotal aspect of riding and driving and directly affects human safety [25]. So, the relatively low selection of using a TT to drive or steer the horse (*n* = 9 TBTs and SBTs combined), is quite surprising. 

The potential physical and psychological harm that TTs can inflict on horses is of concern from a welfare perspective. Previous research has found TTs have a significant negative effect on a horse’s physiological and behavioural state [26]. Findley et al. reported on some of the consequences associated with TT use, with 23% (*n* = 115) of respondents acknowledging some form of physical or behavioural complication [24]. In the current study, 42.9% (*n* = 30) of TT-using respondents had observed some form of physical complication. Interestingly, if a TT was tightened so that the tongue stopped moving (indicating functional tightness), respondents were more likely to report a physical complication. This may indicate that short-term complications (with the most common being redness or bruising or discolouration to the tongue or tongue swelling) are more likely the more tightly a TT is applied. A report by Latimer-Marsh et al. documented a significant increase in behavioural signs of stress, such as head-shaking/tossing, mouth-gaping and backwards ear positioning, as well as physiological signs of stress (specifically elevated salivary cortisol concentrations) [26]. The risks associated with TT use revealed in the current report, in addition to these aforementioned studies, reinforce the perception that TTs are problematic. More detailed investigations into the anatomical effects of the prolonged constriction of vessels within the tongue should be pursued because the aforementioned studies were based largely on anecdotal evidence.

The current study importantly revealed that the duration of TT application significantly increased the likelihood of a respondent reporting a behavioural complication. Of particular concern is that each additional minute a TT was applied increased the odds of reporting a behavioural complication from OR = 1.08, until it approximately doubled at a period of 8–9 min. Given that the average time TTs were left on was longer than this by a factor of 2, TTs used this way would have an even greater likelihood of causing greater stress to the animals. Currently, there are no official guidelines from administrative or governing bodies in any of the major racing jurisdictions on the length of time over which a TT can be applied safely, although Racing Australia stipulates that TTs should not be applied more than 30 min before a race [15]. The findings of the current study, in particular the increased risk of behavioural complications past the 8–9 min threshold highlight the need for further investigation into the effects of a TT on soft tissues such as the vasculature or neural structures found within the tongue, lips or chin. 

If TT use were to be discontinued, then alternative treatments need to be implemented to prevent the occurrence of DDSP in racehorses, since the condition itself may compromise welfare [24]. Tongue-ties are just one of a range of treatments for DDSP in horses [12]. Among the conservative treatments, the use of a “Cornell collar”, grackle or crossed nosebands as well as a variety of bits or bit attachments, that act to depress the tongue, have been described. However, there has been limited scientific study of the efficacy of these items, either in combination or isolation. A recent study reported the Cornell collar, Grackle NBs and figure-of-eight NBs were more effective at preventing DDSP than TTs [13]. The Cornell collar is an external laryngohyoid support device that acts to move the larynx dorsally and rostrally to prevent caudal displacement of the basihyoid bone and subsequent DDSP [25]. Meanwhile, it is proposed that Grackle NBs may help to prevent mouth-opening and reduce palatal instability associated with air entering the space between the tongue and soft palate [25]. In a previous study, Grackle NBs were the most common type of NB reported by racing participants [19] and were also the most common type of NB reported by TT-using respondents in the current study. 

It is important to acknowledge the limitations of the current study. There was a small number of respondents and this may have resulted in some respondent bias. The authors would like to emphasise that the findings of this study should not automatically be applied to the wider horse racing community due to the small sample size. This pilot study is meant primarily as a guide for future researchers. Most respondents were from smaller yards and their responses may not reflect those of trainers from larger enterprises. Attracting the participation of larger trainers would have improved the generalizability of the data. To substantiate the current findings, future research should aim to sample trainers with responsibility for a range of horse numbers. Additionally, the authors acknowledge that there may have been some bias towards certain observations or selections that may be more or less societally acceptable in Australia or New Zealand as many of the current respondents were from these countries. Due to the contentious nature of TTs, there may be some response bias with some respondents having been disinclined to accurately report the risks associated with a commonly used item of gear. On the other hand, advocates of TT use may have been especially likely to respond to the current survey. For these reasons, this should not be considered a definitive global survey but an opportunity to explore relationships among management variables and reported outcomes. 

The survey also failed to differentiate whether respondents were using TTs for specific horses with certain issues or routinely among their horses. As with any survey, reported outcomes may be affected by cognitive dissonance. For example, respondents may wish to believe (and therefore report) that an item of gear achieves what they had hoped, even if it fails to do so. Future studies should more accurately assess the frequency of TT use among trainers as well as whether a TT is being used for a specific purpose in a focal horse. The frequency of respondent observations was not adequately assessed. Conclusions, such as prolonged TT application significantly increasing the likelihood of reporting a behavioural complication, should be noted with caution due to the small sample size. Furthermore, the authors accept that, in the questionnaire, the wording of specific potential complications associated with TT use may have been confusing for some respondents. It should be noted that there is an assumption that respondents who opted to not answer the question on complications related to TT use, did in fact not observe any complications. To avoid having to make this assumption and to refine the data on this topic, future online surveys should deploy logic pathways such that each question must be answered only in either the affirmative or the negative. The authors accept that the distinctions between the three groups of reasons (anatomical, consequential and passive) are not absolute and that some may overlap with one another. However, respondents were not confined to selecting only one reason for using TTs and NBs. 

Bits are a common piece of tack and due to the length of the current survey, were not included so that respondents were not overwhelmed by large numbers of questions. The authors also recommend that future research considers bit use when gathering data on TT use. Finally, we acknowledge that, in a bid to keep the questionnaire brief, the number of questions devoted to the use of TTs was limited. Respondents were unable to report how often they used TTs, whether they used them only for individual horses or on all horses, or whether they used them during training, racing or both. A larger study focused solely on TTs could capture all of this information.

Horse sports are in the public eye more than ever and the improper use of equipment must be discouraged for these sports to maintain their social licence to operate. Racing authorities may see merit in committing to further research that explores the use of TTs and their justification in sports that wish to be considered ethical [27]. As the current report reflects the findings of a pilot study, we encourage those considering further research in these areas to consider its real-world implications. Face-to-face surveying should also be encouraged as it is likely to increase the veracity and number of responses. 

## 5. Conclusions

This pilot study has revealed that respondents are likely to use TTs for reasons related chiefly to improving upper airway issues and preventing the horse’s tongue from moving over the bit. It has revealed that just over half of respondents had encountered either a physical or behavioural complication due to TT use and that complications are associated with the duration of TT use and the way in which it is putatively checked for tightness. Although these preliminary findings cannot be applied to the greater racing community due to the dangers of generalization, this study should inform future researchers in assessing the risks of TTs. 

## Figures and Tables

**Table 1:** The distribution *n* (%) of TT materials reported by respondents. The % reported in each cell represents the number of the type of TT reported over the total TT responses from TBTs (*n* = 62) and SBTs (*n* = 28). An asterisk * denotes a significant difference between the use of a type of TT by either TB or SB respondents. Note that respondents could select more than one type of TT.

Breed	TT Materials					
	Stocking	Elastic	Leather *	Cotton	Cable-Ties	Other
Thoroughbred	45.2%	32.3%	8.1%	11.3%	0	3.2%
(*n* = 62)	(*n* = 28)	(*n* = 20)	(*n* = 5)	(*n* = 7)		(*n* = 2)
Standardbred	21.4%	42.9%	28.6%	0	0	7.1%
(*n* = 28)	(*n* = 6)	(*n* = 12)	(*n* = 8)			(*n* = 2)
Total	48.6%	45.7%	18.6%	10.0%	0	5.7%
	(*n* = 34)	(*n* = 32)	(*n* = 13)	(*n* = 7)		(*n* = 4)

**Table 2:** The reasons for use of TTs nominated by TB (*n* = 47) and SB (*n* = 23) racehorse trainers. A median of 3 reasons was nominated by both TBTs and SBTs.

Reported Reason for TT Use	Thoroughbred (*n* = 47)	Standardbred (*n* = 23)
**Consequential reasons**		
To improve the rider’s/driver’s ability to decelerate the horse	6.4% (*n* = 3)	4.3% (*n* = 1)
To improve the rider’s/driver’s ability to steer the horse	10.6% (*n* = 5)	17.4% (*n* = 4)
To prevent or reduce airway obstruction	72.3% (*n* = 34)	69.6% (*n* = 16)
To prevent or reduce airway noise	55.3% (*n* = 29)	43.5% (*n* = 10)
To improve performance in competition	42.6% (*n* = 20)	39.1% (*n* = 9)
**Anatomical reasons**		
To prevent the horse moving its tongue over the bit	44.7% (*n* = 21)	69.9% (*n* = 16)
**Passive reasons**		
Most people in my sport use them	10.6% (*n* = 5)	8.7% (*n* = 2)
The bit I use requires the use of a tongue-tie	4.3% (*n* = 2)	0
A veterinarian told me that I needed to use one	17.0% (*n* = 8)	17.4% (*n* = 4)
To align with rules of the sport	0	4.3% (*n* = 1)
**Other**	0	4.3% (*n* = 1)

**Table 3:** Distribution of NB use for respondents who never use TTs (*n* = 42) and do use TTs (*n* = 70).

Frequency of NB Use	TT Use	
	No	Yes
Always	38.1% (*n* = 16)	37.1% (*n* = 26)
Usually	21.4% (*n* = 9)	15.7% (*n* = 11)
Sometimes	21.4% (*n* = 9)	18.6% (*n* = 13)
Rarely	11.9% (*n* = 5)	15.7% (*n* = 11)
Never	7.1% (*n* = 3)	5.7% (*n* = 4)

**Table 4:** Agreement of reasons among users (*n* = 61) of both TTs and NBs. Respondents were able to select more than one reason for using both items of gear. Yule-Y coefficient of colligation measures the association between the stated reasons for the use of TTs and NBs.

Reported Reasons	TT Purpose (*n*)	Noseband Purpose (*n*)	Both Used for This Purpose (*n*)	Neither Used for This Purpose (*n*)	Yule-Y Coefficient of Colligation
To improve the rider’s/driver’s ability to decelerate the horse	2	22	2	35	0.23
To improve the rider’s/driver’s ability to steer the horse	3	28	5	25	0.20
To prevent or reduce airway obstruction	28	2	16	15	0.62
To prevent or reduce airway noise	25	2	9	25	0.64
To improve performance in competition	17	6	9	29	0.44
To prevent the horse moving its tongue over the bit	19	6	15	21	0.47
Most people in my sport use them	5	5	1	50	0.33
A veterinarian told me that I needed to use one	8	2	2	49	0.72

## Data Availability

The raw data may be requested through contacting the lead author through email.

## Supplementary Materials

A full transcript of the questionnaire this report is based on is available online at https://www.mdpi.com/2076-2615/11/3/622/s1, File S1: Weller et al 2021 Full Questionnaire: The Reported Use of Tongue-Ties and Nosebands in Thoroughbred and Standardbred Horse Racing—A Pilot Study.
