# Supplementary material for: The Reported Use of Tongue-Ties and Nosebands in Thoroughbred and Standardbred Horse Racing—A Pilot Study
Source: Animals (Basel). 2021 Feb 26;11(3):622. doi: 10.3390/ani11030622 (PMC7996875; doi:10.3390/ani11030622)
Supplement: Supplementary file 1 [file animals-11-00622-s001.pdf]

A transcript of Weller et al.'s 2019-2020 survey. Questions have been highlighted in **bold**. Numbers next to options (e.g. 1 next to racing or 16 next to dressage in the first question) are how RedCAP tracks which answers are given.

Which option best describes your primary activity with horses

1, Racing

15, Camp-drafting/Cutting/Team penning/Working cow horse

16, Dressage

17, Driving

18, Endurance

19, Eventing

20, Mounted games/Horseball

21, Mustering/farm work

22, Natural Horsemanship

23, Polo/Polocrosse

24, Pony Club/Adult riding club/ Working Equitation

25, Show-jumping

26, Showing/hacking

27, Trail-riding/Pleasure riding

28, Western pleasure/ Western Dressage/ Reining/ Barrel racing

30, Pleasure/fun

29, Other

(Other) Please describe your primary

How many horses do you work with?

What is the main breed of horse that you work with currently?

1, Standardbred

2, Thoroughbred

3, American Saddlebred

4, Andalusian/PRE/ Lusitano/Iberian

5, Appaloosa

6, Arabian & Arabian Derivative

7, Australian Riding Pony

8, Australian Stock Horse

9, Brumby

10, Cleveland Bay

11, Clydesdale

12, Cob

13, Coloured Breed

14, Connemara

15, Cross Breed

16, Donkey/Mule

17, Friesian

18, Gypsy Cob

19, Haflinger

20, Irish Draught

21, Irish Sport Horse

22, Lipizzaner

23, Miniature Horse/Pony

24, Morgan

25, Paint

26, Palouse

27, Percheron

28, Peruvian Paso

29, Pinto

30, Pony

31, Quarter Horse

32, Riding Pony

33, Shetland

34, Shire

35, Waler

36, Warmblood

37, Welsh

38, Other

(Other) If the main breed of horses you have in training is NOT in the list above, please specify here.

Do you currently train/race any of your horses with tongue-ties?

1, Yes

2, No

Why not? Please select as many options that relate to your situation.

1, No need

2, Not allowed in the rules of the sport

3, Horse appears uncomfortable/stressed/in pain with a tongue-tie applied

4, Other

How many of your horses currently train/race with a tongue-tie?

What are the most important reasons that underpin your decision to use a tongue-tie?

1, To improve the rider's/driver's ability to decelerate the horse

2, To improve the rider's/driver's ability to steer the horse

3, To prevent or reduce airway obstruction

4, To prevent or reduce airway noise

5, To improve performance in competition

6, To prevent the horse moving its tongue over the bit

7, Most people in my sport use them

8, The bit I use requires the use of a tongue-tie

9, A veterinarian told me that I needed to use one

10, To align with rules of the sport

11, Other

How effective is the tongue-tie at increasing the ability to decelerate the horse?

How effective is the tongue-tie at increasing the ability to steer the horse?

How effective is the tongue-tie at preventing or reducing airway obstruction?

How effective is the tongue-tie at preventing or reducing airway noise?

How effective is the tongue-tie at improving performance in competition?

How effective is the tongue-tie at preventing the horse from moving its tongue over the bit?

1, Extremely effective

2, Very effective

3, Effective

4, Somewhat effective

5, Not at all effective

How do you check that a tongue-tie is fitted correctly? (Tick all that apply)

1, It doesn't slip forward

2, It stops the tongue moving completely

3, The horse cannot remove the tongue-tie

4, The tongue is held within the mouth

5, Other

(Other) Please specify.

Have you seen any of the following complications, in your current or previous horses, immediately or sometime after using a tongue-tie? (Tick all that apply)

1, Tongue swelling during or after application

2, Redness/bruising/discolouration to the tongue

3, Cuts on the tongue

4, Soreness of the tongue or of the lower jaw

5, Nerve damage to the tongue (causing the tongue to hang out of the mouth)

6, Reduced appetite

7, Dropping food

8, Behavioural signs of anxiety or distress

9, Difficulty fastening the tongue-tie

10, Other

(Other) Please specify.

What material are the tongue-ties that you currently use made of? (Tick all that apply)

1, Stocking

2, Elastic

3, Leather

4, Cotton

5, Cable-ties (zippy grips)

6, Other

How long would you usually leave a tongue-tie on a horse during ridden work? (Please give answer in minutes)

Have you ever used any of the following to prevent a horse getting their tongue over the bit? (Tick all that apply)

1, W bit (such as the Serena Song Dexter Racing Bit)

2, Tongue clip/ tongue depressor

3, Miracle bit

4, Winning tongue plate bit

5, Ported bit

6, Bitless bridle

7, Other

(Other) Please specify.

Do you train/race any of your horses while wearing nosebands?

1, Always

2, Usually

3, Sometimes

4, Rarely

5, Never

If not always, why not?

1, No Need

2, Not allowed in the rules of the sport

3, I want my horses to be able to open their mouths

4, Other

How many of your horses are always or usually worked with a noseband?

The following images relate to the question below.

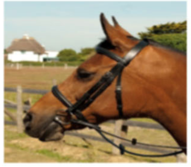

Plain Cavesson Noseband

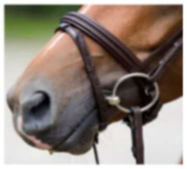

Hanoverian Noseband (With or without Flash)

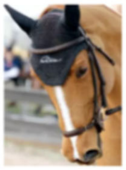

Drop Noseband

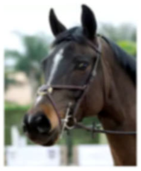

Figure-of-Eight/ Grackle Noseband

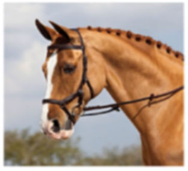

Micklem Noseband

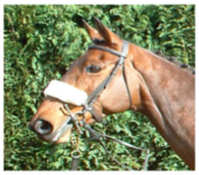

Sheepskin Noseband

Which of the pictures above most closely resembles the noseband you most commonly use?

1, Plain cavesson noseband

2, Hanoverian Noseband (with or without Flash)

3, Drop noseband

4, Figure-of-eight/Grackle noseband

5, Micklem noseband

6, Sheepskin noseband

7, Other

(Other) Please specify.

Does the noseband have a tightening system like the images below

1, Yes

2, No

3, Uncertain

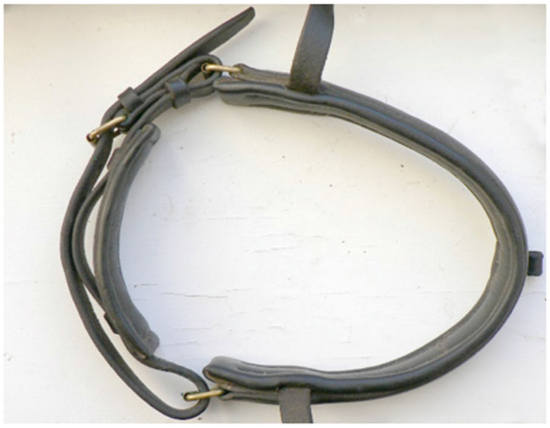

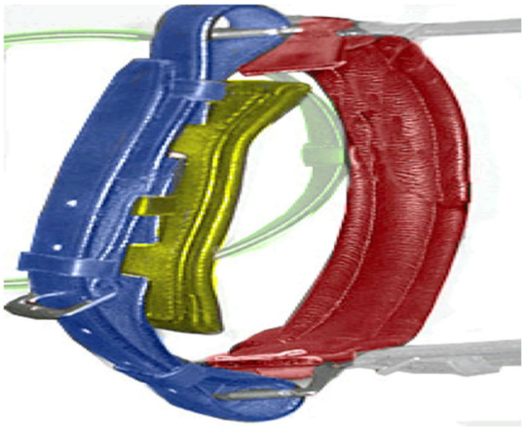

Compared with training your horse at home, do you use tighter nosebands during competition/racing? (Tick the most appropriate response)

1, Always

2, Usually

3, Sometimes

4, Rarely

5, Never

Do you increase the tightness of nosebands after warm-up and immediately before competition? (Tick the most appropriate response)

1, Always

2, Usually

3, Sometimes

4, Rarely

5, Never

Do you usually loosen your horse's noseband for certain activities? (Tick the most appropriate response)

1, Always

2, Usually

3, Sometimes

4, Rarely

5, Never

(If 1-4) Please specify the activities in which you loosen the noseband.

What are the most important reasons that underpin your decision to use nosebands? Please rank up to 5 options that underpin your decision to use nosebands.

1, (a) To improve the rider's/driver's ability to decelerate the horse

2, (b) To improve the rider's/driver's ability to steer the horse

3, (c) To improve the rider's/driver's ability to put the horse on the bit or in a frame/outline

4, (d) To prevent or reduce airway obstruction

5, (e) To prevent or reduce airway noise

6, (f) To improve performance in competition

7, (g) To prevent the horse moving its tongue over the bit

8, (h) To improve the horse's acceptance of the bit/contact

9, (i) To improve the appearance of the horse

10, (j) To prevent the horse opening its mouth

11, (k) To prevent the horse crossing its jaws

12, (l) To prevent the bit sliding through the horse's mouth

13, (m) To align with the rules of the sport

14, (n) The current noseband came with the bridle when I purchased it

15, (o) My instructor/coach/friend told me I needed to use one

16, (p) A veterinarian told me that I needed to use one

17, (q) Most people in my sport use them

18, (r) Other

(Other) Please describe why you use a noseband.

How effective is the type of noseband you use at improving the rider's/driver's ability to decelerate the horse?

How effective is the type of noseband you use at improving the rider's/driver's ability to steer the horse?

How effective is the type of noseband you use at improving the rider's/driver's ability to put the horse on the bit or in a frame/outline?

How effective is the type of noseband you use at preventing or reducing airway obstruction?

How effective is the type of noseband you use at preventing or reducing airway noise?

How effective is the type of noseband you use at improving competition performance?

How effective is the type of noseband you use at preventing the horse from moving its tongue over the bit?

How effective is the type of noseband you use at improving your horse's acceptance of the bit/contact?

How effective is the type of noseband you use at improving the appearance of the horse?

How effective is the type of noseband you use at preventing the horse from opening its mouth?

How effective is the type of noseband you use at preventing the horse from crossing its jaws?

How effective is the type of noseband you use at preventing the bit from sliding through the horse's mouth?

(These questions were answered from a range of:

1, Extremely effective

2, Very effective

3, Effective

4, Somewhat effective

5, Not at all effective)

How long would you usually leave a noseband on a horse during ridden work? Please give your answer in minutes.

Have you seen any of the following complications, in your current or previous horses, either immediately or some time after using a noseband? (Tick all that apply)

1, Hair loss in the area under the noseband

2, Soreness in the area under the noseband

3, Swelling of the area under the noseband

4, Discolouration of the area under the noseband

5, Bleeding from the mouth

6, Lip injuries

7, Reduced appetite

8, Dropping food

9, Behavioural signs of anxiety / distress

10, Head shyness

11, Difficulty bridling the horse

12, Difficulty fastening the noseband

13, Other

(Other) Please specify any other complications.

Do you check the tightness of nosebands? (Tick the most appropriate response)

1, Always

2, Usually

3, Sometimes

4, Rarely

5, Never

Do you test for tightness by checking the space between the noseband and the skin? (Tick the most appropriate response)

1, Always

2, Usually

3, Sometimes

4, Rarely

5, Never

Where on the horse's head would you usually check the tightness of the noseband?

1, At the bridge of the nose

2, Along the right or left cheek

3, Under the chin

4, Other

(Other) please specify.

Which of the following would you consider sufficient space under the noseband?

1, 0 fingers can be inserted under the noseband

2, 1 finger can be inserted under the noseband

3, 2 fingers can be inserted under the noseband

4, More than 2 fingers can be inserted under the noseband

Do you ever use a taper gauge (like the one illustrated below) to check noseband tightness?

1, Yes, I have used taper gauges and find them useful

2, Yes, I have used taper gauges and do not find them useful

3, No, I have never used a taper gauge

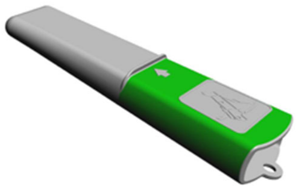

What is your current country of residence

1, Antigua and Barbuda

2, Australia

3, The Bahamas

4, Barbados

5, Belize

6, Canada*

7, Dominica

8, Grenada

9, Guyana

10, Ireland

11, Jamaica

12, New Zealand

13, St Kitts and Nevis

14, St Lucia

15, St Vincent and the Grenadines

16, Trinidad and Tobago

17, United Kingdom

18, United States of America

20, Sweden

19, Other

(Other) Please specify.

(If Australia) What state do you live in?

1, New South Wales

2, Victoria

3, Queensland

4, Western Australia

5, South Australia

6, ACT

7, Tasmania

8, Northern Territory

What age group do you fall into?

1, 18-25

2, 25-35

3, 36-45

4, 46-55

5, 56-65

6, 66-75

7, 76-85

8, 86-95

9, 96+

What is your gender?

1, Male

2, Female

3, Other

How many years have you worked with horses?

1, 0-5

2, 6-10

3, 11-15

4, 16-20

5, 21-25

6, 26-30

7, 31-35

8, 36-40

9, 41-45

10, 46-50

11, 51-55
